# Supplementary figures and images for: Genome-Wide Analysis and Expression of the GRAS Transcription Factor Family in Avena sativa
Source: Genes (Basel). 2023 Jan 6;14(1):164. doi: 10.3390/genes14010164 (PMC9858933; doi:10.3390/genes14010164)

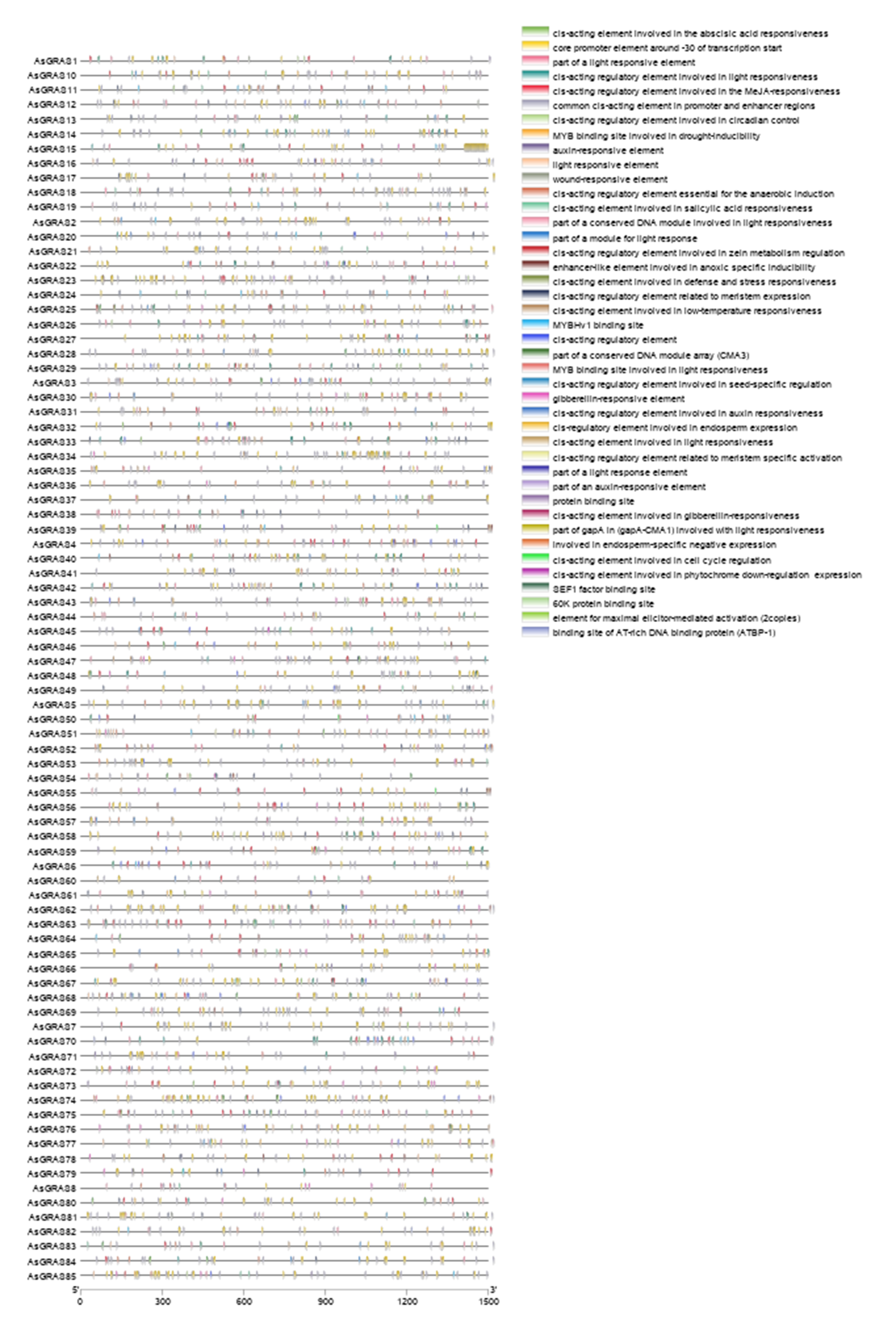

Supplement: Supplementary file 1 [file genes-14-00164-s001.zip › Fig. S1.png]
